# Ceramide Synthase 6: Comparative Analysis, Phylogeny and Evolution

**DOI:** 10.3390/biom8040111

**Published:** 2018-10-08

**Authors:** Roger S. Holmes, Keri A. Barron, Natalia I. Krupenko

**Affiliations:** 1 Griffith Research Institute for Drug Discovery and School of Environment and Science, Griffith University, Nathan, QLD 4111, Australia; 2 Department of Nutrition, UNC-Chapel Hill, UNC Nutrition Research Institute, Kannapolis, NC 28081, USA; kab154@email.unc.edu

**Keywords:** ceramide synthase, *CerS6*, enzymes, genes, human, mouse, vertebrates, invertebrates, yeast

## Abstract

Ceramide synthase 6 (*CerS6*, also known as *LASS6*) is one of the six members of ceramide synthase gene family in humans. Comparisons of CerS6 amino acid sequences and structures as well as of *CerS6* gene structures/locations were conducted using data from several vertebrate genome projects. A specific role for the *CerS6* gene and protein has been identified as the endoplasmic reticulum C_14_- and C_16_-ceramide synthase. Mammalian CerS6 proteins share 90–100% similarity among different species, but are only 22–63% similar to other CerS family members, suggesting that *CerS6* is a distinct gene family. Sequence alignments, predicted transmembrane, lumenal and cytoplasmic segments and N-glycosylation sites were also investigated, resulting in identification of the key conserved residues, including the active site as well as C-terminus acidic and serine residues. Mammalian *CerS6* genes contain ten exons, are primarily located on the positive strands and transcribed as two major isoforms. The human *CERS6* gene promoter harbors a large CpG island (94 CpGs) and multiple transcription factor binding sites (TFBS), which support precise transcriptional regulation and signaling functions. Additional regulation is conferred by 15 microRNA (miRNA) target sites identified in the *CERS6* 3′-UTR region. Phylogenetic analysis of the vertebrate *CerS1–6* gene families relationships supports a major role for the CerS6 enzyme that is strongly conserved throughout vertebrate evolution.

## 1. Introduction

Mammalian ceramide synthase (*CerS*) genes were initially discovered by the homology search of human genome using the Lag1p motif from the yeast *LAG1* gene [1]. In yeast, *LAG1* was shown to regulate the life span [2] and support ceramide biosynthesis [3], and as such its homolog *LAC1* and other homologous genes were dubbed *LASS*es (longevity associated genes). These genes were found in most species varying in numbers, with both mice and humans having six paralogs: *LASS 1–6* [4]. Further studies of the mammalian *LASS* genes function revealed that the key feature of the proteins encoded by these genes is the ability to synthesize ceramide, and consequently the nomenclature was changed to *CerS 1–6* [4]. Importantly, each of the ceramide synthases was shown to utilize a subset of acyl-CoAs, thus producing ceramides with specific acyl chain length [5,6]. Ceramides play a central role in sphingolipid metabolism and function as important components defining biophysical properties of cellular membranes [7]. In the past decades, distinct roles of ceramides as signaling molecules and as mediators of apoptosis and inflammatory responses have also been established [5,6,8]. It should be noted, that though CerSes generate different ceramide species, their genes show similarities in sequence and structure, and the corresponding protein products display similar domain organization, catalytic properties and subcellular localization. 

At the same time, different ceramide synthases show different biological properties and play vastly diverse roles in numerous biological processes [5]. CerS1 catalyzes the synthesis of C_18_ acyl chain ceramides and plays a major role in the development of brain and other neural tissue [1,9]. CerS2 preferentially catalyzes the synthesis of C_20_–C_26_ acyl chain ceramides which are essential during development. Accordingly, *CerS2* null mice were reported as having chronic alterations of both liver and brain physiology with increased hepatocyte apoptosis and turnover, chronic and progressive myelin degeneration as well as membrane material accumulation in lysosomes [10,11]. CerS3 is predominantly expressed in testis and skin and exhibits a broad acyl chain preference synthesizing C_26_–C_32_ acyl chain ceramides. In agreement with very specific function of this enzyme, *CerS3* null mice die soon after birth due to the loss of skin barrier function [12,13]. CerS4, which is specific for C_18_–C_22_ acyl-CoA, is found at high levels in the skin epidermis, heart, skeletal muscle, liver, lung and white adipose tissue and may play a role in regulating stem cell homeostasis, with *Cers4* null mice exhibiting major reductions in C_20_ acyl chain ceramide levels [14,15]. CerS5 is the major ceramide synthase in lung epithelial cells and in the white and grey matter of brain [5]. It is essential for maintaining C_16_ ceramide pools and may contribute to the development of diet induced obesity [16,17].

The last member of the mammalian ceramide synthase family, CerS6 was first reported by Weinmann et al. [18] in 2005. This enzyme generates C_14_- and C_16_-ceramides and phylogenetically is most closely related to CerS5 [5]. *Cers6* knockout mice demonstrated a significant decrease in C_16_-containing sphingolipids that was accompanied by behavioral abnormalities [19], protection from high-fat diet-induced obesity and glucose intolerance [20], as well as protection from colitis [21,22] and neutrophils activation [23]. Moreover, *CerS6* expression was required for optimal T cell activation, proliferation and cytokine production in response to alloantigen and for subsequent induction of graft-versus-host disease [24]. Additionally, activation of CerS6 and subsequent elevation of C_16_-ceramide were demonstrated in response to folate stress [25], serum starvation [26], deficiency of cytochrome c oxidase [27] and methotrexate [28]. Despite high homology and identical substrate preference of this enzyme to CerS5, which to some extent has been studied with regard to its membrane organization and structural basis of specificity [29,30], the structure and function of CerS6 are still not well investigated. In the present study we used bioinformatic and phylogenetic methods to examine the structures of vertebrate *CerS6* genes and enzymes and their evolutionary relationship with the other *CerS* genes as well as ancestral yeast and invertebrate genes.

## 2. Results and Discussion

### 2.1. Comparison of Vertebrate CerS6 Amino Acid Sequences and their Relation to Human CERS1–5 Sequences

Examination of the non-redundant protein sequence databases for several vertebrate genomes using basic local alignment search tool (BLAST) analyses for CerS6 amino acid sequence, as well as BLAST-like alignment tool (BLAT) analyses of the predicted CerS6 sequences using the University of California, Santa Cruz (UCSC) genome browser allowed us to predict locations for these genes, including exon boundary locations and gene sizes. Comparison of predicted gene and protein characteristics to their human and mouse counterparts is presented in Table 1. The human and other mammalian CerS6 sequences examined were 90% or more identical, suggesting that these are the products of the same family of genes.

**Figure 1:**
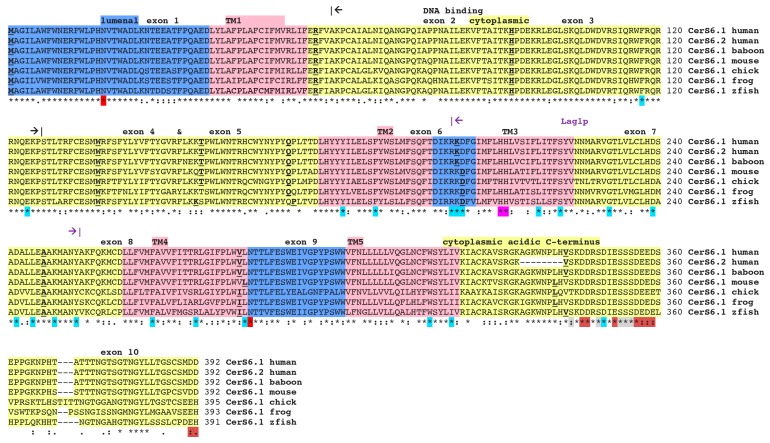
Amino acid sequence alignments for vertebrate CerS6 enzymes: See Table 1 for sources of sequences; * shows identical residues for polypeptides; - similar alternate residues; - dissimilar alternate residues; exon start sites are underlined and in bold; predicted lumenal sites are shown in blue; predicted transmembrane sequences are in pink and numbered TM1–5 from N-terminal end; predicted cytoplasmic regions are in yellow; a predicted homeobox DNA binding domain (residues 66–127) is shown by black arrows; the Lag 1p motif (residues 202–253) is denoted by violet arrows; two predicted N-glycosylation sites in lumenal regions are shown in red; the two adjacent histidine residues (His) in the active site are in magenta; the acidic C-terminal domain is indicated, with acidic amino acid residues marked in brown and C-terminal serine (Ser) residues subject to phosphorylation shown in grey; 21 conserved residues for all CerS1–6 and yeast LAC1 sequences are shown in cyan; zfish refers to the zebra fish sequence. Note differences in sequences between human CERS6.1 and CERS6.2 isoforms. Sequence alignment has been completed using CLUSTAL Omega (EMBL-EBI, Hinxton, Cambridgeshire, UK, https://www.ebi.ac.uk/Tools/msa/clustalo/).

**Figure 2:**
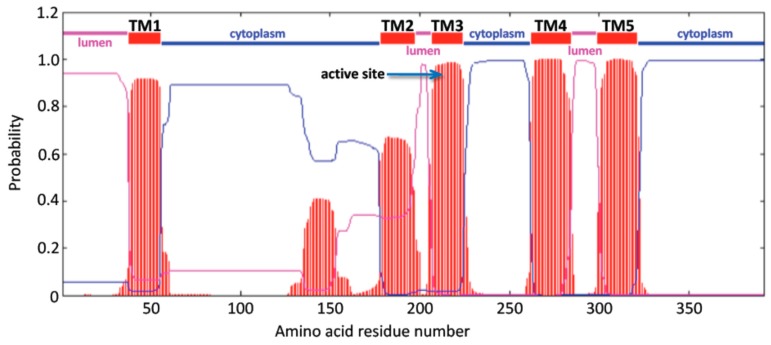
Predicted transmembrane organization for human CERS6 enzyme. Regions of hydrophobicity are shown as striped red segments, with five transmembrane domains (TM1–5) identified (TMHMM Server v. 2.0, DTU Bioinformatics, Kongens Lyngby, Denmark). Predicted lumenal and cytoplasmic regions are shown in magenta and blue lines; the His211-His212 residues of the active site were positioned within TM3. *Y*-axis: probability (0–1) of forming a transmembrane helix; *X*-axis: amino acid position within human CERS6.

All primate CerS6 sequences included 392 amino acid residues and were more than 99% identical to human protein. The other eutherian mammalian CerS6 sequences showed the identity to human enzyme of 95–99% and the polypeptide chains were 391–396 amino acid residues long. Alignment of two human transcripts [18,19] baboon (*Papio anubis*), mouse [19], chicken (*Gallus gallus*), frog (*Xenopus tropicalis*) and zebra fish (*Danio rerio*) CerS6 protein sequences are shown in Figure 1. Baboon, chicken, frog, and zebra fish sequences were deduced by our analyses. Enzyme topology with predicted major domains determined using TMHMM Server v.2.0 [31,32] is shown in Figure 1 and Figure 2. 

**Figure 3:**
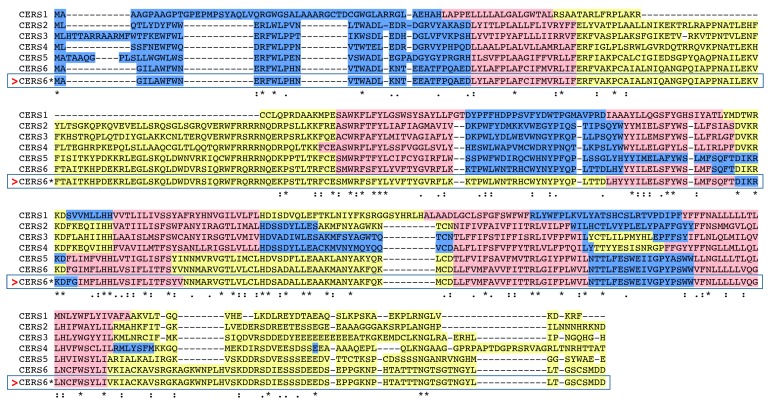
Amino acid sequence alignment of human CERS enzymes. Sequence sources are listed in Table 1 and Table 3. The PSI/TM-Coffee software was used to align sequences and map membrane domains. Predicted lumenal sites are shown in blue; predicted transmembrane sequences are in pink and predicted cytoplasmic regions are in yellow; *, shows identical residues for all polypeptides; : - similar alternate residues; . - dissimilar alternate residues. Rectangular frame shows the same CerS6 sequence with the alternative domain topology prediction (TMHMM Server 2).

Our analysis suggests that CERS6 is an integral membrane protein and has three domains located within the lumen of the endoplasmic reticulum (residues 1–37, 199–206, 285–302), five transmembrane regions (designated TM1–5, residues 38–55, 178–198, 207–224, 262–284, 303–323, correspondingly) and three cytoplasmic regions (residues 56–177, 225–261, 324–392). Such domain organization coincides with the predictions made earlier using different bioinformatic tools, TopPredII and UniProt [30,33]. Currently, no experimental evidence for the precise topology of CerS6 has been presented, and no crystal structures for any of the CerS enzymes exist. Thus, it cannot be excluded that the precise topological arrangements of CERS6 may differ from the predicted here. Indeed, alignment of all six human ceramide synthases using PSI/TM-Coffee software predicted six transmembrane domains for CERS6 and quite different distribution of lumenal and cytoplasmic regions (shown in Figure 3). The TMHMM Server v.2 analysis of CERS6 (Figure 1) is included for comparison in the same alignment (rectangular frame) and demonstrates the first cytoplasmic region that is 42 amino acids longer, as well as lumenal orientation of the region homologous to the second cytoplasmic domain of CERSes 1–4. Similar controversy was observed in the membrane topology studies of CERS5 [29]. Four out of fifteen software algorithms used for the topology analyses predicted five transmembrane domains for CERS5, while eleven other predicted six transmembrane domains. It should also be noted that PSI/TM-Coffee analysis (T-Coffee Server, Centre for Genomic Regulation, Barcelona, Spain) indicated direct connection of lumenal and cytoplasmic domains for CERS1, CERS2, CERS3 and CERS6, which are sterically improbable. Experimental interrogation of the mammalian CerSes topology, similar to that published for yeast CerS enzymes [34] could help resolve the prediction inconsistencies.

CerS6 sequence alignment (Figure 1) and analysis have identified a number of specific amino acid residues conserved among the vertebrates: the 52 amino acid conserved sequence (Lag1p motif) shown to be important for the ceramide synthase activity [1,34,35]; the two adjacent histidine residues (His211 and His212) located within TM3 and also important for CerS CERS activity (first reported for CerS1 [35]); Lys134, mutation of which resulted in a 50% loss of activity, as well as residues comprising a functional Hox domain region (66–127) of which 12 terminal residues were shown to be important for enzyme activities in CerS5 and CerS6 only [36]. Recently a 150 residue region in the TLC domain of CerS enzymes (amino acids 159–309 for CerS5) has been identified as a determinant of substrate specificity [29]. Additionally, two putative N-glycosylation sites located within predicted lumenal domains (18NVT, 285NTT) were identified, similar to the previous work where experimental evidence of glycosylation of the N18 only was obtained [30]. Furthermore, C-terminal serine residues were identified as potential sites for CerS6 phosphorylation: S336, S341, S345, S346 and S347. Phosphorylation of these residues has been reported previously for CerS2–6 and was shown to contribute to the maintenance of catalytic activities [37] as well as stimulate Lac1/Lag1 activity and formation of complex sphingolipids in yeast (inositol-phosphate containing) [38]. 

Additionally, sequence comparison revealed several groups of distinct conserved residues the function of which has not been defined yet. Among these are the three tyrosine’s, 180–182, located within the second transmembrane domain and the strictly conserved stretches of acidic amino acids in C-terminus including Asp346, Asp347, Asp350, Glu352, Asp356, Glu357, Glu358 and Asp359 (Figure 1) which may be important for the CerS6 carboxyl-terminus function within the cytoplasm. Additional residues with unknown function found to be conserved not only between the vertebrate CerS6 sequences but in nematode (LAGR1 and HYL1) and yeast LAC1 sequences as well, are: Phe117 and Glu124 in the first cytoplasmic domain; Tyr182 and Phe188 in TM2; Arg202-Lys203-Asp204 in the second lumenal region; Leu218 and Ser222 located within TM3; Gly231, Asp239, Asp242, Leu244, Lys.249 and Tyr253 in the second cytoplasmic segment; Phe267, Arg275 and Leu284 in TM4; as well as Trp318 and Ile322 in TM5. The future structure-function studies will explain the reason for their conservation in CerS6 evolution.

In agreement with high overall sequence similarity of CerS6 enzymes, comparisons of theoretical isoelectric points for eutherian mammalian proteins showed that a theoretical isoelectric point (pI) value of 8.0 was consistent for all of these enzymes, with the exception of baboon CerS6 (pI = 7.1) (Table 1). This difference is apparently explained by the substitution of a basic amino acid (Lys154, pI = 9.03) in human CERS6 for an acidic Glu154 (pI = 5.03) in baboon enzyme. 

### 2.2. CERS6 Gene Expression

Two major transcripts have been identified for *CERS6*, (*CERS6.1* and *CERS6*.2), with the second isoform encoding an enzyme with 384 residues, lacking eight amino acids (Ala335–His342) within the C-terminal cytoplasmic domain, but retaining key active site and transmembrane sequences. Interestingly, only one transcript has been identified previously for CERS6 [33], and the properties and function of the short form identified here are not known at present. The shorter *CerS6.2* was routinely observed for all mammalian and chicken BLAST analyses undertaken, suggesting that two *CerS6* isoforms were retained throughout mammalian evolution. 

**Figure 4:**
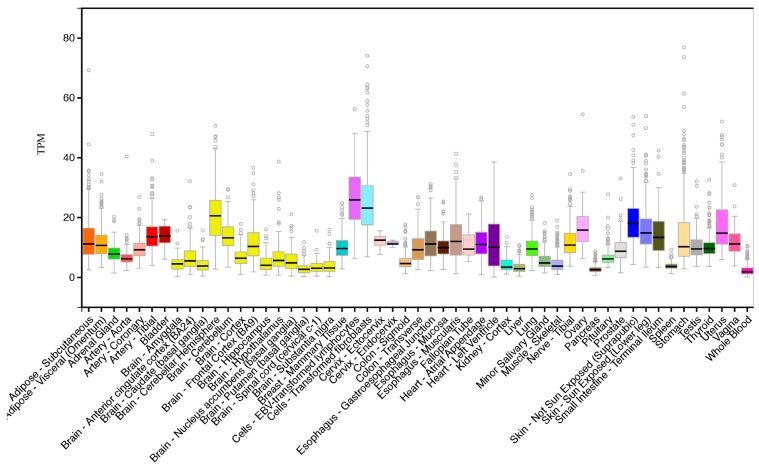
Comparative tissue expression levels for human *CERS6.* RNA-seq gene expression profiles across 53 selected tissues (or tissue segments) were obtained from the public database for human *CERS6*, based on expression levels for 175 individuals (Data Source: GTEx Portal, GTEx Analysis Release V6p (dbGaP, accession number phs000424.v6.p1; https://gtexportal.org/home/gene/ENSG00000172292.10). TPM, Transcripts per million, calculated from a gene model with isoforms collapsed to a single gene. Box plots show a median and 25th and 75th percentiles; points are shown as the outliers if they are above or below 1.5 times the interquartile range.

Tissue expression analysis of human *CERS6* transcripts demonstrated a wide distribution profile, with highest levels observed in transformed lymphocytes and fibroblasts, as well as in skin, small intestine, cerebellar hemispheres and frontal cortex, heart ventricle, ovary, stomach and uterus (Figure 4). This is indicative of a broad role throughout the body for this enzyme in the biosynthesis of C_14_ and C_16_ ceramides that are required for maintaining normal membrane function and fluidity and for variety of signaling processes [39].

### 2.3. Comparative Analysis of Vertebrate CerS6 Genes and Human CERS1–5 Genes

**Figure 5:**
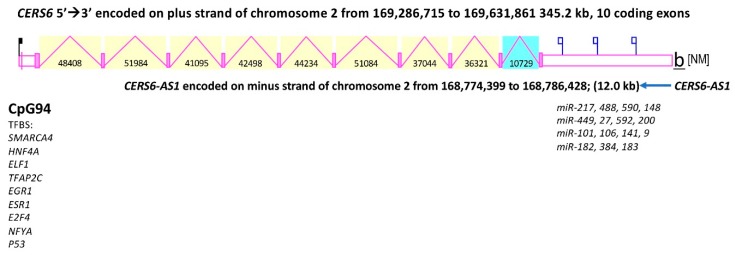
Structure of the human *CERS6* gene. Derived from the AceView website [40]; the major isoform variant is shown with capped 5′- and 3′-ends for the predicted mRNA sequences; introns (pink lines) and exons (pink boxes) are shown; the length of the messenger RNA (mRNAs) (as kilobases or kb) are shown; a CpG island (CpG94) is shown for the *CERS6* promoter; 15 miRNA binding sites were identified for the 3′UTR of the human *CERS6* gene; the direction for transcription is shown; TFBS refers to transcription factor binding sites located within the *CERS6* promoter; *CERS6-AS1* refers to an antisense gene located proximal to *CERS6*.

Table 1 summarizes comparisons of chromosomal locations, exonic structures and sizes for vertebrate *CerS6* genes. The human *CERS6* gene is located on the positive strand of human chromosome 2, contains ten exons, and spans > 300 Kbps of DNA (*CERS6* gene structure is depicted in Figure 5). It is the largest of the human *CERS* genes ranging from 9.8 Kbps (*CERS2*), 27.6 Kbps (*CERS1*), 37.6 Kbps (*CERS5*), 50 Kbps (*CERS4*), 143.9 Kbps (*CERS3*) to 313.2 Kbps (*CERS6*) (see Table 1 and Table 2). Interestingly, the mammalian *CerS6* genes are 3–5 times larger than the bird, lizard, frog or fish *CerS6* genes, though the number of exons and amino acids are preserved (Table 1). The human *CERS* genes are located on separate chromosomes, with the exception of *CERS1* and *CERS4* which are distantly located on human chromosome 19 (Table 1 and Table 2), consistent with previous report [5]. While human *CERS1* gene has seven exons, the other human *CERS*es contain nine (*CERS2*) or ten (*CERS3*, *CERS4*, *CERS5*, *CERS6*) exons. All of the mammalian *CerS6* genes examined also contained ten coding exons, were larger in size (>243 Kbps for rat *Cers6*) than the other human *CERS* genes and were encoded on the positive DNA strand (with the exception of marmoset *CerS6*) (Table 1). 

Compared to human CERS6 protein, other human ceramide synthases had slightly varying lengths: CERS1 (350 amino acids, aa), CERS2 (380 aa), CERS3 (383 aa), CERS4 (394 aa) and CERS5 (392 aa) (Table 1 and Table 2). The human and other mammalian CerS6 sequences examined were 90% or more identical, suggesting that these are the products of the same family of genes. However, pair-wise comparisons of sequence identities of the human CERS6 and human CERS1–5 proteins showed identities within 22–63% range (Table 3), indicating that these orthologs are members of distinct *CerS* gene families (Table 1 and Table 2). The human CERS5 and CERS6 sequences were 62% identical, while the identity to human CERS1–4 enzymes was lower, indicating that CERS5 and 6 are more closely related to each other than to other human CERS enzymes, as previously reported [18]. The human CERS1 sequence was more distantly related with other human CERS homologs (22–27% identity) whereas human CERS2 and CERS3 sequences showed more similarity with each other (51% identity) than with other human CERSes (Table 3). The sequence alignment (Figure 3) of all six human CERS enzymes demonstrates that the first cytoplasmic domain and the last 30 amino acid stretch of the c-terminal cytoplasmic domain show the lowest identity between the family members, while the transmembrane and lumenal domains have higher identities. 

Interestingly, a *CERS6-AS1* (antisense) gene was located proximate to human *CERS6* on the minus strand of chromosome 2 (Figure 5). The non-coding transcription is not rare in biological systems: it takes place in organisms from all kingdoms of life and 25–40% of human expressed sequences are connected to antisense transcription [41,42]. Natural antisense transcripts were shown to function in early embryonic development, healthy growth and disease. For example, p53 tumor suppressor was shown to be regulated by the anti-sense transcript Wrap53 [43]. Thus, presence of *CERS6-AS1* indicates that it may play a role in regulating expression of *CERS6* in different tissues of the body or during development. Figure 5 also depicts the exonic and intronic structure of the human *CERS6* gene, together with the presence of a CpG island containing 94 CpGs and several transcription factor-binding sites within the untranslated 5′ region, as well as binding sites for 15 microRNAs within the 3′ untranslated region of this gene. 

It should be noted, that Figure 5 shows the regulatory elements (CpG island, transcription factors and miRNAs) identified by the on-line tools, which may not reflect all regulatory elements. Thus, recent study from our laboratory has experimentally demonstrated that human CERS6 is transcriptionally regulated by the p53 protein via direct binding to a non-canonical site in the CERS6 promoter region [44]. It is apparent that with one experimentally established and eight predicted transcription factor binding sites, a CpG island and 15 miRNA-binding sites, the *CERS6* gene has multiple mechanisms for regulation of its transcription and translation. Unfortunately, most of these mechanisms which may play significant roles during development or stress response are not studied. 

### 2.4. Evolution of Vertebrate CerS1–6 Genes from the Invertebrate Lagr1 and Hyl1 Genes

**Figure 6:**
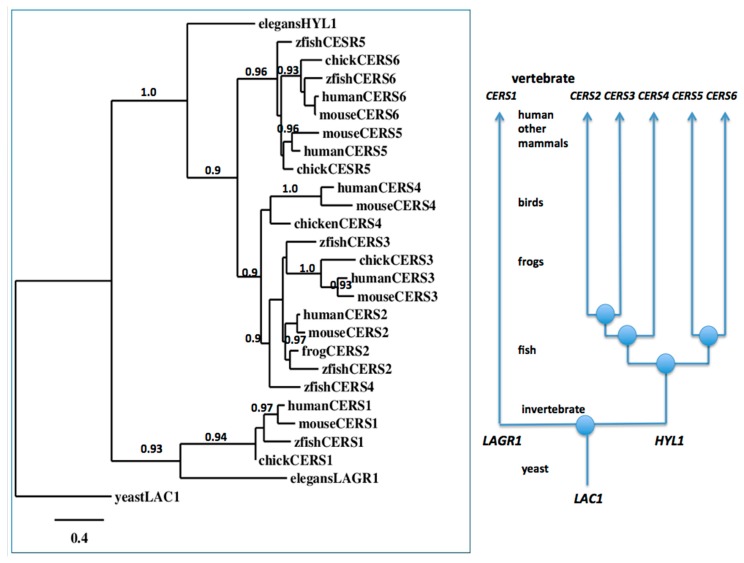
Phylogenetic tree of vertebrate CerS1-6 amino acid sequences and nematode LAGR1 and HYL1 sequences rooted with the yeast Lac1 sequence. The tree is labelled with the ceramide synthase names and the names of the animal or organism and is rooted with the yeast (*Saccharomyces cerevisiae) LAC1* sequence. See Table 1 and Table 2 for details. Note the clusters corresponding to the *CerS1–6* gene families. A genetic distance scale is shown. The number of times a clade (sequences common to a node or branch) occurred in the bootstrap replicates are shown. Replicate values of 0.9 or more, which are highly significant, are shown with 100 bootstrap replicates performed in each case. A proposed sequence of gene evolution events is shown arising from ancestral yeast *LAC1* and invertebrate *LAGR1* and *HYL1* genes.

A phylogenetic tree (Figure 6) was generated by the progressive alignment of four vertebrate CerS6 sequences, several vertebrate CerS1–5 amino acid sequences and nematode (*Caenorhabditis elegans*) Hyl1 and Lagr1 sequences, with the baker’s yeast (*Saccharomyces cerevisiae*) Lac1 sequence [34], which was also used to root the tree. Details of the vertebrate, nematode and yeast *CerS-like* genes and enzymes are presented in Table 1 and Table 3. Interestingly, the rat *CerS5* gene is located on X chromosome, a feature that has not been reported previously. The phylogram (Figure 6) shows clustering of the CerS-like sequences into two groups, which is consistent with their evolutionary relatedness and separate groupings as distinct CerS1-like and CerS2–6-like genes and enzymes. It is apparent that the *CerS1* gene evolved from a distinct invertebrate *CerS*-like gene family LAGR1 which has been retained throughout vertebrate and invertebrate evolution. In contrast, the invertebrate *HYL1*-like gene family has undergone several gene duplication events prior to the appearance of the ancestral vertebrate genome, forming the *CerS2–6* gene families, also retained throughout vertebrate evolution. 

Among these gene families, *CerS5*/*CerS6* and *CerS2*/*CerS3* gene families are more closely related to *CerS4* than to each other. It is also apparent that the evolution of the mammalian *CerS6* genes has occurred at a more conservative rate than of the mammalian *CerS5* genes, given the reduced genetic distances observed for the primate *CerS6* encoded sequences, in comparison with the primate *CerS5* encoded sequences. Stronger conservation of the *CerS6* gene family could reflect an important additional role of the enzyme, considering a very similar acyl-CoA specificity of CerS5 and CerS6. The phylogenetic tree also suggests that the invertebrate *CerS5* gene may have served as a primordial ancestor for both of the vertebrate *CerS5* and *CerS6* genes. 

## 3. Methods

### 3.1. CerS Gene and Protein Identification 

Protein BLAST analyses used National Center for Biotechnology Information (NCBI) web tools [45] to examine vertebrate and invertebrate homologs using human and mouse CerS6 amino acid sequences previously described (Table 1) [18,46]. Non-redundant protein sequence databases for the following genomes were examined using the BLASTP algorithm: chimpanzee (*Pan troglodytes*), gorilla (*Gorilla gorilla*), gibbon (*Nomascus leucogenys*), baboon (*Papio anubis*), rhesus monkey (*Macaca mulata*), marmoset (*Callithrix jacchus*), mouse lemur (*Miscrocebus murinus*), mouse (*Mus musculus*), rat (*Rattus norvegicus*), horse (*Equus caballus*), dog (*Cannis familiaris*), opossum (*Monodelphis domestica*), chicken (*Gallus gallus*), lizard (*Anolis carolensis*), frog (*Xenopus tropicalis*) and zebrafish (*Danio rerio*). The sequences for predicted mRNAs and encoded CerS6-like proteins were recorded and retained in FASTA format. To further study each of the *CerS6* genes (predict exon boundary locations and gene sizes (Table 1)), BLAST-like alignment tool (BLAT) analyses using the UCSC Genome Browser [47] were undertaken for each of the predicted CerS6 amino acid sequences. Default settings were applied in each case. Similar BLAT analyses were performed for other ceramide synthases. Structures for the two major human CerS6 splice-variants were obtained using the AceView website and NCBI web tools [40]. CerS6 sequence alignment was completed using CLUSTAL Omega web tools with default settings (EMBL-EBI, Hinxton, Cambridgeshire, UK, https://www.ebi.ac.uk/Tools/msa/clustalo/).

### 3.2. Predicted Structures and Properties of Mammalian CerS6 Enzymes 

Molecular weights, isoelectric points and predicted location of transmembrane, lumenal and cytosolic domains for mammalian and chicken CerS6 proteins were obtained using Expasy web tools [31]. The identification of conserved domains for CerS6 proteins was conducted using NCBI web tools [32]. Analysis of CerS6 domain organization was performed using TMHMM Server v. 2.0 from DTU Bioinformatics (Kongens Lyngby, Denmark, http://www.cbs.dtu.dk/services/TMHMM/). Alignment of human Cers1–6 enzymes and prediction of the membrane, cytoplasmic and lumenal regions in all human CerS enzymes was performed using PSI/TM-Coffee V.11.00.d625267 software (T-Coffee Server, Centre for Genomic Regulation, Barcelona, Spain) with default settings [48]. 

### 3.3. Comparative Human Tissue CERS6 Gene Expression

RNA-seq gene expression profiles across 53 selected tissues (or tissue segments) were obtained from the public database for human genes, based on measured expression levels of *CERS6* in samples from 175 individuals (GTEx Consortium, 2015). Data Source: GTEx Analysis Release V6p (dbGaP phs000424.v6.p1; https://gtexportal.org).

### 3.4. Phylogeny Studies and Sequence Alignments

Phylogenetic analyses were performed to reconstruct phylogenetic relationships using free phylogeny.fr software (CNRS, France, http://www.phylogeny.fr/index.cgi) with default settings [49,50] (Figure 7). Sequences were identified as vertebrate and invertebrate CerS-like proteins and aligned using Clustal Omega [51] (Table 1)*.*

## 4. Conclusions

The results of the present study indicate that vertebrate *CerS6* and *CerS5* genes and encoded proteins represent two closely related *CerS* gene families, which are distinct from other vertebrate *CerS* gene families (*CerS1–4*), similar to previously reported analyses [5,6]. At least eleven domains were identified for the vertebrate CerS6 proteins examined: three lumenal domains, including an N-terminus lumenal domain containing a conserved N-glycosylation site (Asn18); five predicted transmembrane domains (TM1–5) define organization of this enzyme within the endoplasmic reticulum membrane; and three cytoplasmic domains, with the C-terminus containing conserved acidic residues, that may define the function of this domain in cytoplasm. The active site for CerS6 has not been precisely experimentally defined yet, however conserved amino acids, including a double histidine site (His211-His212) located within TM3; Lys134, mutation of which resulted in a 50% loss in activity [36] and three consecutive tyrosine residues (Tyr180-Tyr181-Tyr182) may serve as elements contributing to the biosynthetic or other key roles for this enzyme. 

Mammalian CerS6 enzymes are encoded by a single large gene (>240 Kbps) in all genomes studied, with ten exons located on the positive strand for all mammalian genes examined, with the exception of marmoset *CerS6*, for which the ten exons were located on the negative strand (Table 1). Phylogenetic studies using vertebrate CerS6 sequences, vertebrate CerS1–5 sequences and nematode LAGR1 and HYL1 sequences, indicated that these genes and enzymes represent two distinct but related gene families which have apparently evolved from a single yeast *LAC1* gene. 

## Figures and Tables

**Table 1:** Vertebrate *CerS6* genes and proteins.

Gene	Organism	Species	Chromosome ^ location	Coding Exons (strand)	Gene Size bps	GenBank ID *	UNIPROT ID	Amino Acids	Subunit MW (pI)	Similarity to Human CerS6, %
*CERS6*	Human	*Homo Sapiens*	2:168,456,249–168,775,134	10 (+strand)	313,211	NM_001256126	Q6ZMG9-2	392	45,794 (8.0)	100
*CerS6*	Chimp	*Pan troglodytes*	2B:172,816,690–173,137,732	10 (+strand)	321,043	* XP_001154789	H2RCH7	392	45,794 (8.0)	100
*CerS6*	Gorilla	*Gorilla gorilla*	2B:60,802,122–61,119,872	10 (+strand)	317,751	* XP_004032792	A0A2I2YTR2	392	45,794 (8.0)	100
*CerS6*	Gibbon	*Nomascus leucogenys*	22A:58,027,154–58,354,057	10 (+strand)	321,406	* XP_004092010	G1QX90	392	45,767 (8.0)	99.7
*CerS6*	Baboon	*Papio anubis*	12:30,381,688–30,694,736	10 (+strand)	313,049	* XP_017802114	A0A2I3NGN0	392	45,824 (7.1)	99.9
*CerS6*	Rhesus monkey	*Macaca mulatta*	12:55,419,737–55,731,333	10 (+strand)	311,597	* XP_014965738	> H9Z7B4	392	45,824 (8.0)	99.5
*CerS6*	Green monkey	*Chlorocebus sabaeus*	10:53,972,990–54,294,221	10 (+strand)	321,232	* XP_007963450	A0A0D9RS37	392	45,797 (8.0)	99.2
*CerS6*	Marmoset	*Callithrix jacchus*	6:49,862,083–50,206,291	10 (−strand)	344,209	* XP_002749346	F7I442	392	45,812 (8.0)	99.0
*CerS6*	Mouse lemur	*Microcebus murinus*	^ KQ055119v1:198,292–459,448	10 (+strand)	261,157	* XP_012637321	na	392	45,736 (7.1)	99.0
*CerS6*	Mouse	*Mus musculus*	2:68,861,636–69,108,591	10 (+strand)	246,956	NM_001347161	H3BL08	392	45,717 (8.0)	95.4
*CerS6*	Rat	*Rattus norvegicus*	3:55,094,637–55,338,445	10 (+strand)	242,941	* XP_006224534	F1LTP8	392	45,749 (8.0)	95.2
*CerS6*	Horse	*Equus caballus*	18:48,176,490–48,465,346	10 (+strand)	288,857	NM_001301183	> F7BVU2	392	45,773 (8.0)	97.4
*CerS6*	Dog	*Canis familiaris*	36:13,308,109–13,602,677	10 (+strand)	294,569	> * XP_025315848	F1P6P4	391	45,585 (8.0)	95.4
*CerS6*	Opossum	*Monodelphis domestica*	4:177,901,296–178,232,211	10 (+strand)	330,304	* XP_001375412	K7E682	396	46,206 (8.5)	90.0
*CerS6*	Chicken	*Gallus gallus*	7:18,442,947–18,552,421	10 (−strand)	109,475	* XP_025008400	E1C4X9	387	45,079 (8.50)	77.6
*CerS6*	Lizard	*Anolis carolinensis*	1:245,388,446–245,518,415	10 (−strand)	129,970	* XP_008124100	G1K9E6	386	44,737 (8.8)	75.5
*CerS6*	Frog	*Xenopus tropicalis*	9:63,975,031–64,070,810	10 (+strand)	93,133	* XP_004917763	>F6ZS80	393	46,096 (8.5)	78.8
*CerS6*	Zebrafish	*Danio rerio*	9:49,172,637–49,239,755	10 (−strand)	67,119	* XP_693283	F1QPF3	391	45,860 (8.6)	79.5

UNIPROT IDs (Swiss Institute of Bioinformatics, http://kr.expasy.org) and GenBank IDs (NIH, Bethesda, MD, USA, http://www.ncbi.nlm.nih.gov/genbank/) provide the sources for the gene and protein sequence data; gene sizes are given as base pairs of nucleotides; +strand and −strand refer to the transcribed strand; exons refer to coding exons; MW refers to predicted molecular weight; pI refers to theoretical isoelectric point; * predicted gene sequences; ^ chromosome location not available; > sequence is slightly different from another database sequence.

**Table 2:** Vertebrate, nematode and yeast *CerS*-like genes and proteins.

Gene	Organism	Species	Chromosome ^ Location	Coding Exons (strand)	Gene Size bps	GenBank ID *	UNIPROT KD	Amino Acids	Subunit MW (pI)
*CERS1*	Human	*H. sapiens*	19:18,868,552-18,896,072	7 (−strand)	27,593	NP_067090	P27544	350	39,536 (9.2)
*CerS1*	Mouse	*M. musculus*	8:70,315,775–70,331,587	7 (+strand)	15,812	NP_619588	P27545	350	40,100 (8.7)
*CerS1*	Chicken	*Gallus gallus*	28:3,502,179–3,512,634	7 (+strand)	10,456	NP_001264694	F1N9S0	354	41,235 (9.2)
*CerS1*	Zebrafish	*D. rerio*	22:4,791,880–4,847,724	7 (+strand)	55,845	* XP_009294228	F1Q5B1	358	42,229 (5.5)
*CERS2*	Human	*H. sapiens*	1:150,965,173–150,975,003	10 (−strand)	9830	NM_022075	Q96G23	380	44,876 (9.0)
*CerS2*	Mouse	*M. musculus*	3:95,320,063–95,322,789	10 (+strand)	2727	NM_001320492	Q924Z4	380	45,024 (8.9)
*CerS2*	Chicken	*G. gallus*	25:2,627,873–2,630,268	10 (−strand)	2396	* XP_003642714	> A0A1D5PKE9	377	44,621 (8.9)
*CerS2*	Zebrafish	*D. rerio*	16:1,360,026–1,370,907	10 (+strand)	10,882	* XP_693668	Q90YY7	383	45,191 (8.6)
*CERS3*	Human	*H. sapiens*	15:100,400,395–100,544,286	10 (−strand)	143,892	NM_001290342	Q8IU89	383	46,316 (7.6)
*CerS3*	Mouse	*M. musculus*	7:66,733,234–66,823,692	10 (+strand)	90,459	* XP_011249179	Q1A3B0	383	46,081 (8.3)
*CerS3*	Chicken	*G. gallus*	10:17,297,468–17,326,587	10 (−strand)	29,120	* XP_424275	F1N8P2	380	45,302 (8.9)
*CerS3*	Zebrafish	*D. rerio*	7:9,904,627–9,961,727	10 (+strand)	35,391	* XP_002662790	A0A140LFW4	380	45,066 (7.2)
*CERS4*	Human	*H. sapiens*	19:8,209,353–8,262,421	10 (+strand)	50,069	NM_024552	Q9HA82	394	46,399 (9.2)
*CerS4*	Mouse	*M. musculus*	8:4,493,405–4,526,079	10 (+strand)	32,675	NM_026058	Q9D6J1	393	46,017 (8.7)
*CerS4*	Chicken	*G. gallus*	28:100,066–130,610	10 (−strand)	30,545	* XP_015155290	A0A1D5P159	398	47,408 (8.5)
*CerS4*	Zebrafish	*D. rerio*	22:4,707,655–4,733,106	10 (+strand)	25,452	NM_153670	Q90YY6	406	47,636 (8.4)
*CERS5*	Human	*H. sapiens*	12:50,129,798–50,167,422	10 (−strand)	37,625	NM_147190	Q8N5B7	392	45,752 (8.2)
*CerS5*	Mouse	*M. musculus*	15:99,735,592–99,772,515	10 (−strand)	36,924	NM_028015	Q9D6K9	414	48,167 (8.4)
*CerS5*	Chicken	*G. gallus*	^268,433–285,586	10 (+strand)	17,154	* XP_424486	A0A1D5NZ43	391	45,516 (8.9)
*CerS5*	Zebrafish	*D. rerio*	22:5,758,094–5,821,733	10 (−strand)	63,640	NM_199628	A8E7D4	387	45,541 (9.2)
*LAGR1*	Nematode	*Caenorhabditis elegans*	I:13,724,671–13,731,990	7 (−strand)	7317	NM_061002	Q9XWE9	360	42,743 (8.5)
*HYL1*	Nematode	*C. elegans*	IV:8,540,663–8,544,055	8 (+strand)	2958	NM_069058	G5ED45	368	43,851 (8.9)
*LAC1*	Yeast	*Saccharomyces cerevisiae*	XI:427,295–428,551	1	1256	NM_001179574	P28496	418	48,992 (9.5)

UNIPROT IDs (Swiss Institute of Bioinformatics, http://kr.expasy.org) and GenBank IDs (NIH, Bethesda, MD, USA, http://www.ncbi.nlm.nih.gov/genbank/) provide the sources for the gene and protein sequence data; gene sizes are given as base pairs of nucleotides; +strand and −strand refer to the transcribed strand; exons refer to coding exons; pI refers to theoretical isoelectric points; * predicted gene sequences; ^ chromosome location not available; > sequence slightly different from another database.

**Table 3:** Sequence identities (%) for human CERS1–6 enzymes. Three distinct human CERS sub-families were identified based on amino acid sequence comparison: CERS1, (22–27%); CERS2–4, (37–51%); and CERS5–6, (62%).

Human CerSes	CerS1, Identity (%)	CerS2, Identity (%)	CerS3, Identity (%)	CerS4, Identity (%)	CerS5, Identity (%)	CerS6, Identity (%)
CerS1	100	27	25	31	27	29
CerS2	27	100	53	49	41	41
CerS3	25	53	100	45	40	39
CerS4	31	49	45	100	43	42
CerS5	27	41	40	43	100	63
CerS6	19	41	39	42	63	100
